# Comparative Analysis of Alcohol and Aldehyde Dehydrogenase Activities in Prostate Cancer and Benign Prostatic Hyperplasia

**DOI:** 10.3390/jcm15187042

**Published:** 2026-09-11

**Authors:** Adam Rafał Nowiński, Wojciech Jelski, Marta Skrodzka, Barbara Mroczko, Mariusz Gryko, Karolina Orywal

**Affiliations:** 1 Department of Urology, Independent Public Health Care Center of the Ministry of the Interior and Administration in Białystok, Fabryczna 27, 15-471 Białystok, Poland; 2 Department of Biochemical Diagnostics, Faculty of Pharmacy with the Division of Laboratory Medicine, Medical University of Bialystok, Waszyngtona 15A, 15-269 Białystok, Poland; wojciech.jelski@umb.edu.pl (W.J.); mroczko@umb.edu.pl (B.M.); 3 Department of Urology, St. George’s University Hospital, London SW17 0QT, UK; marta.skrodzka@stgeorges.nh-s.uk; 4 Department of Surgical Nursing, Medical University of Bialystok, 15-274 Białystok, Poland; mariusz.gryko@umb.edu.pl; 5 1st Clinical Department of General and Endocrine Surgery, Medical University of Bialystok Clinical Hospital, 15-276 Białystok, Poland

**Keywords:** prostate cancer, alcohol dehydrogenase, aldehyde dehydrogenase, enzyme activity, isoenzymes, prostate metabolism, benign prostatic hyperplasia, biomarkers

## Abstract

**Background:** Prostate cancer (PCa) remains one of the most frequently diagnosed cancers in men worldwide, and there is growing interest in the role of metabolic reprogramming in its development. Key metabolic pathways include alcohol dehydrogenase (ADH) and aldehyde dehydrogenase (ALDH), which are enzymes involved in the oxidation of alcohols and aldehydes, the detoxification of xenobiotics, and the regulation of cell proliferation and the oxidative stress response. Despite well-documented changes in enzyme activity in other malignancies, their activity in prostate tissue has not yet been thoroughly investigated. This study aimed to evaluate the activity of total ADH and its isoenzymes (classes I–IV), and ALDH classes I and III, in prostate cancer tissue compared with benign prostatic hyperplasia (BPH). **Methods:** Enzyme activities were determined using spectrophotometric and fluorometric methods in prostate tissue obtained from 48 patients with PCa (26 with low-risk and 22 with intermediate-risk disease) and from 42 patients with benign prostatic hyperplasia (BPH). The study groups were compared using non-parametric statistical tests, with *p* < 0.05 considered statistically significant. **Results:** The isoenzyme ADH class III showed the highest activity in both studied groups. The activity of ADH class I was significantly higher in PCa tissue compared with BPH (*p* = 0.0499), while ALDH I and III activity was also significantly higher in PCa tissue than in BPH (*p* = 0.0007). No significant differences were observed in total ADH or its other isoenzymes. Significant correlations were found among some ADH isoenzymes, but not with ALDH. **Conclusions:** Increased activity of class I ADH and ALDH enzymes in prostate cancer tissue may reflect disease-related metabolic changes and suggest their potential significance in the biology of prostate cancer. These findings provide new insights into metabolic reprogramming in prostate cancer and support the need for further studies to determine the biological and potential clinical significance of these enzymes.

## 1. Introduction

Prostate cancer (PCa) is one of the most commonly diagnosed cancers in men worldwide, particularly in developed countries. According to GLOBOCAN 2020, approximately 1.4 million new cases of prostate cancer and 375,000 deaths were estimated worldwide in 2020 [1]. Consequently, some European countries have launched new programs or trials to reconsider the benefits of broad PC screening. These initiatives include updated strategies that go beyond prostate-specific antigen (PSA) testing, the incorporation of magnetic resonance imaging, and targeted screening for men with high genetic risk [2]. Autopsy studies have shown that prostate cancer prevalence increases significantly with each decade of age. While the disease is rarely detected in men under 30 (5%), it reaches almost 60% in men over 79 [3]. Differences in prostate cancer prevalence have also been reported among men of Asian, European, and African descent. Latent prostate cancer has been detected in approximately 50% of Asian men by age 90, in Caucasian men by age 80, and in men of African descent by age 60 [4].

Various factors are considered in the pathogenesis of prostate cancer, such as genetic, epigenetic, hormonal, inflammatory, and metabolic mechanisms. Clinical trial results suggest that healthy lifestyle modifications, including plant-based diets, have a favorable influence before and after prostate cancer treatment [5]. Androgen-dependent transcriptional activation of the androgen receptor (AR) is central to prostate tumorigenesis, as it regulates genes that control cell proliferation, survival, and tumor progression [6]. In addition to genomic alterations, non-genomic mechanisms, particularly epigenetic modifications, have emerged as key regulators of prostate cancer development. These include histone modifications, DNA methylation, and non-coding RNA regulation, all of which play a crucial role in prostate tumor formation [7]. Additionally, chronic inflammation, oxidative stress, and dysregulation of cell cycle and apoptosis pathways contribute to carcinogenesis [8,9,10]. In recent years, increasing attention has been paid to metabolic reprogramming in prostate cancer, including alterations in lipid metabolism, glycolysis, and redox balance [11]. Metabolic adaptations are increasingly recognized as important determinants of prostate cancer progression and treatment resistance [12]. Fatty acid metabolism is extensively rewired during prostate cancer development, with alterations occurring both systemically and locally within the tumor microenvironment [13]. These metabolic changes may be associated with cellular adaptation to a microenvironment characterized by metabolic and oxidative stress [14,15].

Among the numerous metabolic alterations associated with carcinogenesis, abnormalities in alcohol dehydrogenase (ADH) and aldehyde dehydrogenase (ALDH) activities have gained increasing attention. These enzymes play an important role in the oxidative metabolism of alcohols and aldehydes, contribute to the detoxification of xenobiotics, and regulate cell proliferation, differentiation, and the cellular response to oxidative stress [16,17]. Previous studies suggest that altered activity of ADH, its isoenzymes, and ALDH has been observed in various types of cancer tissue, including liver, breast, and gastrointestinal tract [18]. These enzymes may participate in the initiation and progression of malignancies by influencing retinoic acid biosynthesis and detoxifying toxic aldehydes, such as acetaldehyde. Acetaldehyde combines with cellular compounds, disturbing cell physiology and resulting in tumor development [19,20]. The potential role of ADH in prostate carcinogenesis may vary depending on the specific class of isoenzymes, as they exhibit distinct substrate specificities and catalytic properties. In addition to ethanol oxidation, ADH enzymes participate in the metabolism of endogenous aldehydes and retinoids, processes that can influence redox homeostasis and cell differentiation. Consequently, alterations in the activity of specific ADH isoenzymes may play a significant role in neoplastic transformation and tumor progression [16,18]. Notably, ALDHs have been identified as markers of cancer stem cells and are associated with tumor aggressiveness and therapy resistance in many types of cancer [21]. Additionally, TGFB1/MMP11 signaling contributes to the ALDH1A1-driven PCa metastases [22].

However, despite evidence of altered ADH and ALDH activity in various malignancies, no comprehensive studies have been conducted on their enzymatic activity in prostate cancer. In particular, limited data are available on the activity of individual ADH isoenzymes and ALDH classes in PCa tissue and their relationship with disease risk. The aim of this study was therefore to determine and compare the activity of class I–IV ADH isoenzymes, total ADH, and ALDH class I and III in prostate cancer tissues. By identifying tumor-specific enzymatic patterns, we aimed to contribute to a better understanding of tumor biology and the metabolic differences between PCa and BPH and to examine whether these enzyme activities are associated with differences between low- and intermediate-risk PCa.

## 2. Materials and Methods

### 2.1. Material

Specimens of cancerous prostate tissue (80–100 mg) were obtained from 48 cancer patients (mean age 70 years, range 49–87 years) during radical prostatectomy. The patients were divided into two groups: 26 were diagnosed with low-risk prostate cancer, and 22 with intermediate-risk PCa. The diagnoses were based on the 2025 European Association of Urology (EAU) guidelines (Table 1).

None of the patients had undergone chemotherapy or radiotherapy before tissue collection. The control group comprised benign prostatic hyperplasia tissue obtained during electrosurgical resection of the prostate from 42 patients (mean age 68 years, range 48–88 years). Tissue fragments were selected from the central or deeper portions of the resected tissue rather than from the cut or visibly cauterized surface. Whenever possible, areas showing visible coagulation or thermal damage were avoided. BPH samples were used as a non-malignant comparative group, as healthy prostate tissue is rarely available for research purposes; this approach is commonly adopted in prostate cancer studies [24,25]. All patients had a history of occasional alcohol consumption. Patients with a history of liver disease, major metabolic disorders, or treatment with medications known to affect ADH/ALDH activity were excluded from the study. The research protocol was approved by the local institutional Bioethical Committee (Approval No. APK.002.102.2021, dated 25 February 2021). Informed consent for participation was obtained from all subjects involved in the study.

### 2.2. Methods

#### 2.2.1. Preparation of Tissue Extract

All specimens were immediately frozen at −80 °C after collection and stored at −80 °C until enzyme activity analysis. The tissue samples to be examined (1:4 *w*/*v*) were homogenized in a potassium phosphate buffer (0.1 M; pH 7.4) for 20 s using an ultrasonicator (Sonoplus HD 70, Bandelin, Berlin, Germany). The samples were then centrifuged at 14,000 rpm for 30 min at 4 °C. The supernatant was used to determine the ADH and ALDH activities. Laboratory investigators were not blinded to the clinical group allocation during enzymatic activity measurements.

#### 2.2.2. Determination of Total ADH Activity

Total ADH activity was estimated using a separate photometric assay with p-nitrosodimethylaniline (NDMA; Sigma Diagnostics, St. Louis, MO, USA) as the substrate [26]. Because this assay differs from those used for determination of individual ADH isoenzymes in terms of substrate and detection method, the resulting numerical values are not directly comparable or additive. The reaction mixture (2 mL) consisted of 0.1 mL of supernatant, 1.8 mL of a 26 μM solution of the substrate in 0.1 M sodium phosphate buffer at pH 8.5, and 0.1 mL of a mixture containing 0.25 M n-butanol (Avantor Performance Materials Poland S.A., Gliwice, Poland) and 5 mM NAD. Reduction of NDMA was monitored at 440 nm using a Shimadzu UV/VIS 1202 spectrophotometer (Shimadzu Europa GmbH, Duisburg, Germany).

#### 2.2.3. Determination of Class I and III ALDH Activity

Classes I and III of aldehyde dehydrogenase isoenzyme activity were measured using a fluorogenic method, which is based on the oxidation of 6-methoxy-2-naphthaldehyde (Sigma Diagnostics, St. Louis, MO, USA) [27]. The reaction mixture contained: supernatant, 60 μL of substrate, 20 μL of 11.4 mM NAD^+^ (Sigma Diagnostics, St. Louis, MO, USA), and 2.8 mL of 50 mM sodium phosphate buffer solution (pH 8.5). The mixture also contained a 12 mM solution of 4-methylpyrazole (Sigma Diagnostics, St. Louis, MO, USA) as a specific inhibitor of ADH activity. The fluorescence was read at an excitation wavelength of 310 nm and an emission wavelength of 360 nm using a Shimadzu RF-5301 spectrofluorometer (Shimadzu Europa GmbH, Duisburg, Germany). This fluorometric assay measures the combined enzymatic activity of ALDH isoenzymes belonging to classes I and III and should therefore be interpreted as the combined activity of these two classes.

#### 2.2.4. Determination of Class I and II ADH Isoenzymes

Classes I and II of ADH isoenzyme activity were measured using fluorogenic substrates (4-methoxy-1-naphthaldehyde for class I and 6-methoxy-2-naphthaldehyde for class II, Sigma Diagnostics, St. Louis, MO, USA) in a reduction reaction, according to Wierzchowski et al. [28]. The assays were performed in a reaction mixture containing a supernatant (60 μL), 150 μL substrate (300 μM), 100 μL of NADH (1 mM, (Sigma Diagnostics, St. Louis, MO, USA), and 2.69 mL of 0.1 M sodium phosphate buffer solution at pH 7.6 (2.69 mL). The measurements were performed on a Shimadzu RF-5301 spectrofluorometer using an excitation wavelength of 316 nm for both substrates, with an emission wavelength of 370 nm for class I and 360 nm for class II isoenzymes.

#### 2.2.5. Determination of Class III ADH Isoenzyme

The assay mixture for class III alcohol dehydrogenase contained: supernatant (100 μL), caprylic alcohol (31 μL of 1 mM, Sigma Diagnostics, St. Louis, MO, USA) as a substrate, NAD (240 μL at 1.2 mM, Sigma Diagnostics, St. Louis, MO, USA), and 0.1 M NaOH-pyrophosphate buffer at pH 8.0 [29]. The final volume was 2 mL. Reduction of NAD was monitored at 340 nm, and the reaction was carried out at 25 °C on a Shimadzu UV/VIS 1202 spectrophotometer.

#### 2.2.6. Determination of Class IV ADH Isoenzyme

The assay mixture for class IV ADH activity contained a supernatant (50 μL), m-nitrobenzaldehyde (132 μL of 80 μM, Sigma Diagnostics, St. Louis, MO, USA), and NADH (172 μL of 86 μM, (Sigma Diagnostics, St. Louis, MO, USA) in a 0.1 M sodium phosphate buffer at pH 7.5 [30]. The oxidation of NADH was monitored at 340 nm at 25 °C using a Shimadzu UV/VIS 1202 spectrophotometer.

#### 2.2.7. Protein Assays

Protein concentration was measured using the Lowry method with bovine serum albumin as the standard (Sigma Diagnostics, St. Louis, MO, USA) [31], and enzyme activities were normalized to protein concentration and expressed as nmol/min/mg of protein.

#### 2.2.8. Statistical Analysis

Statistica 13.3 (TIBCO Software Inc., Palo Alto, CA, USA) was used to perform statistical analysis of results. Data distribution was assessed using the Shapiro–Wilk test. As the variables were not normally distributed, non-parametric tests were applied. The Mann–Whitney U test was used to evaluate differences between the groups, while the Spearman rank correlation test was applied to examine the relationship between variables. For comparison among the three groups (BPH, low-risk PCa, and intermediate-risk PCa), the Kruskal–Wallis ANOVA test was performed, followed by post-hoc pairwise comparisons. No formal post-hoc test was performed. No formal correction for multiple comparisons was applied. The 95% confidence intervals (CI) for the medians were calculated using the asymptotic method based on the standard error of the median. Statistically significant differences were considered results with *p* < 0.05. Potential outliers were assessed based on the distribution of the data. No observations were excluded solely because of extreme values.

## 3. Results

The activity of ADH, its isoenzyme classes I-IV, and ALDH class I and III in prostate cancer and benign prostatic hyperplasia tissue is shown in Table 2.

We demonstrated the presence of ADH and ALDH activities in prostate cancer and benign prostatic hyperplasia tissue. Comparing ADH isoenzyme activities revealed that class III ADH exhibited the highest activity. The median activity of this isoenzyme was 6.174 nmol/min/mg of protein in prostate cancer tissue and 7.696 nmol/min/mg of protein in BPH tissue. In cancer tissue, class III ADH showed the highest activity among the ADH classes measured, followed by class IV, class II, and class I. A nominally significant difference was observed for ADH I activity (*p* = 0.0499); however, this finding was not confirmed by the Kruskal–Wallis analysis. The median activity of this class in the cancer group was 0.060 nmol/min/mg of protein, compared with 0.052 nmol/min/mg of protein in BPH tissue. Analysis of the other ADH isoenzyme classes did not reveal any significant differences. Total ADH activity did not differ significantly between groups (PCa: 0.558 nmol/min/mg of protein; BPH: 0.438 nmol/min/mg of protein). Notably, ALDH class I and III activity was significantly higher in the PCa group (0.242 nmol/min/mg of protein) than in the BPH group (0.103 nmol/min/mg of protein), with a highly statistically significant difference (*p* = 0.0007). To further assess the magnitude of between-group differences, rank-biserial correlation coefficients (r_rb) were calculated. The effect size was small for ADH I-IV and total ADH activity, whereas a larger effect was observed for ALDH activity (r_rb = 0.406).

**Figure 1:**
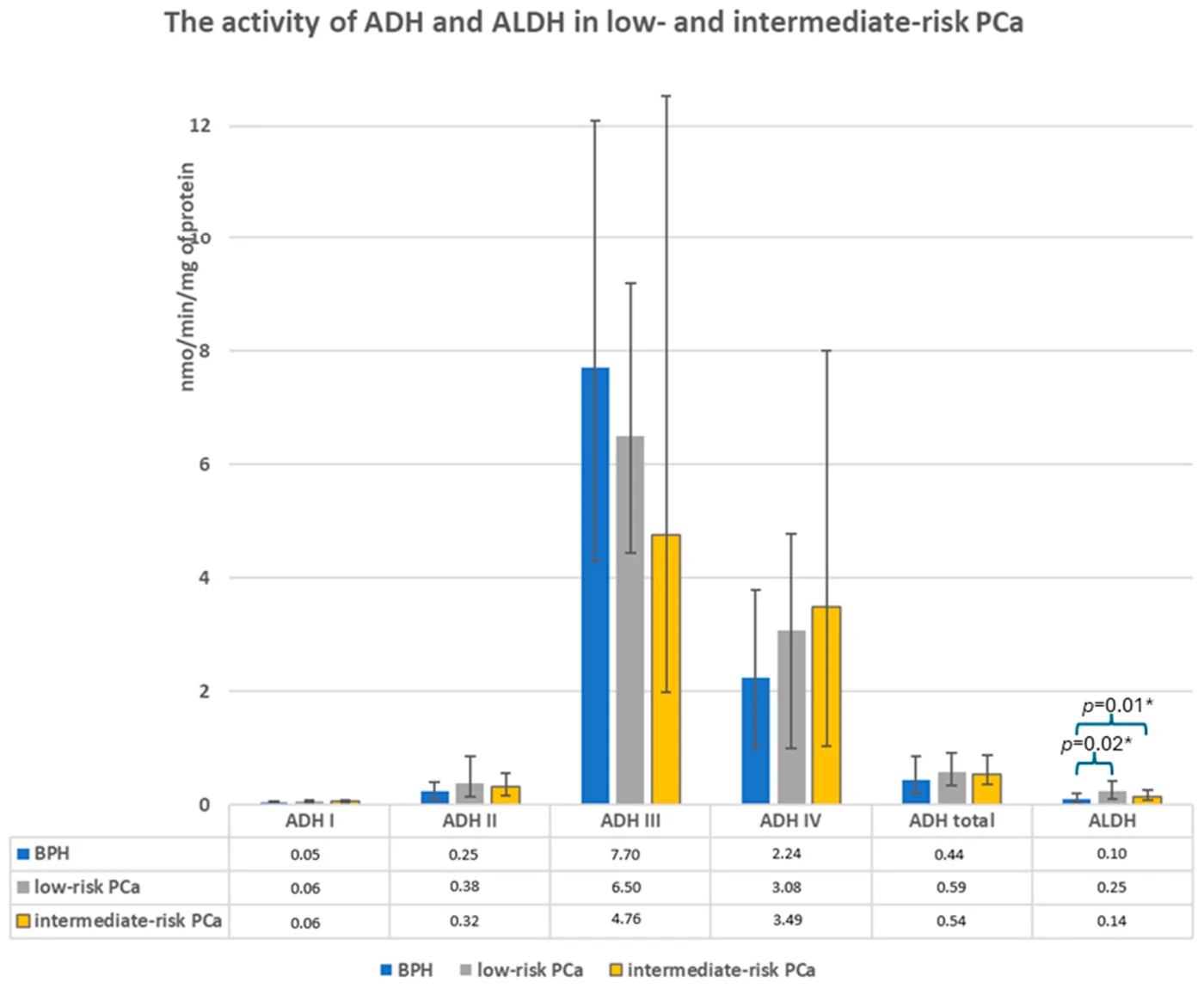
The activity of ADH and ALDH in low-risk and intermediate-risk prostate cancer and BPH. Data are presented as median values with interquartile ranges (IQR). Statistically significant differences between the groups are indicated by an asterisk (*). BPH *n* = 42, low-risk PCa *n* = 26, intermediate-risk PCa *n* = 22.

Figure 1 demonstrates the comparison of ADH and ALDH isoenzyme activity in low- and intermediate-risk prostate cancer. The highest activity was observed for ADH III across all groups. The highest activity was observed in the BPH group, followed by the low-risk prostate cancer group (6.50 nmol/min/mg of protein) and the intermediate-risk PCa group (4.76 nmol/min/mg of protein). The intermediate-risk PCa group exhibited slightly higher ADH IV activity (3.49 nmol/min/mg of protein) than the low-risk PCa group (3.08 nmol/min/mg of protein) and the BPH group (2.24 nmol/min/mg of protein). Statistically significant differences were found only in class I and III ALDH activity: BPH exhibited significantly lower ALDH I and III activity than both the low-risk and intermediate-risk PCa groups (*p* = 0.02 and *p* = 0.01, respectively).

The correlations between the activities of the tested enzymes are shown in Table 3. All statistically significant correlations were weak, with the highest correlation coefficients observed between ADH I and ADH III (r = 0.317, *p* = 0.002), between ADH I and ADH IV (r =0.305, *p* = 0.003), and between ADH I and total ADH activity (r = 0.245, *p* = 0.017). ADH II was significantly correlated with ADH IV (r = 0.273, *p* = 0.007), and ADH III was significantly correlated with ADH IV (r = 0.287, *p* = 0.005). The highest correlation coefficients were observed between ADH I and ADH III (r = 0.317) and between ADH I and ADH IV (r = 0.305). The lowest statistically significant correlation was found between ADH I and total ADH activity (r = 0.245, *p* = 0.017). Other correlations with ALDH were weak and not statistically significant. The observed correlations between ADH isoenzymes should be interpreted with caution because enzyme activities were normalized to protein concentration obtained from the same tissue supernatant, which may contribute to correlations between variables. Therefore, these findings should not be interpreted as evidence of coordinated regulation.

Additional correlation analyses were performed to investigate the associations between ADH activity and clinicopathological parameters in PCa patients (Table 4). A significant negative correlation was observed between ADH III activity and Gleason score (r = −0.374, *p* = 0.008). No significant correlations were found between the activities of ADH I, ADH II, ADH IV, total ADH, and ALDH I and III and Gleason score. Furthermore, no significant correlations were observed between ADH activities and TNM stage, risk group, or PSA levels.

## 4. Discussion

A growing body of evidence suggests that the metabolism of cancer cells is fundamentally different from that of noncancerous tissues. This study provides the first comprehensive comparison of the activity of alcohol dehydrogenase isoenzymes (classes I–IV), total ADH, and aldehyde dehydrogenase (classes I and III) in prostate cancer tissue and benign prostatic hyperplasia. Our findings demonstrate that both ADH and ALDH are present in prostate tissue, with selected enzymes showing significantly different activities between malignant and benign conditions. We observed that the activity of ADH class I was significantly higher in PCa tissue compared with BPH. Our findings differ from those of Stamis et al., who reported downregulation of ADH1B at the transcriptional level in prostate cancer tissues, suggesting a potentially protective regulatory role for this isoenzyme [32]. This discrepancy may be related to differences in the level of biological regulation assessed, as our study measured enzymatic activity whereas Stamis et al. examined ADH1B transcription. Differences in tissue characteristics, patient populations, and experimental methodology may also contribute to the divergent findings. mRNA expression does not always correlate directly with protein level or enzymatic function. Gygi et al. found that identical mRNA levels can correspond to up to 20-fold differences in protein amount, and constant protein levels can occur with even 30-fold differences in mRNA levels [33]. Furthermore, the dysregulation of gene expression characteristic of cancer cells often involves changes in the stability and efficiency of translation of existing mRNAs, enabling the rapid adaptation of the protein profile conducive to proliferation and a competitive advantage over normal cells [34]. Zhang et al. found that the majority of the differences in levels of expression occur at the protein level, with no corresponding change at the mRNA level, implying the importance of post-transcriptional regulatory mechanisms [35]. Furthermore, the ADH I activity measured in our study reflects the combined activity of several class I ADH isoforms (ADH1A, ADH1B, and ADH1C), rather than just ADH1B alone. These three isoforms have a high degree of sequence homology but differ in terms of their substrate specificity, kinetic properties, and tissue expression. The most well-known function of ADH class I is its involvement in the conversion of ethanol to acetaldehyde in the liver, but this enzyme also participates in the oxidation of retinol to retinal, a substrate for retinoic acid biosynthesis [36,37]. These findings suggest that since class I ADH is involved in retinol metabolism and acetaldehyde production, its higher activity may reflect altered retinoid signaling or oxidative stress responses during tumorigenesis in the prostate gland.

In contrast, the highest enzymatic activity across all studied groups was observed for class III ADH, with the highest activity in BPH tissues, followed by low- and intermediate-risk PCa. This isoenzyme is primarily associated with the metabolism of formaldehyde and endogenous lipid peroxidation products, suggesting that it may play a role in maintaining redox homeostasis [38]. Although not statistically significant, the decrease in ADH III activity in intermediate-risk PCa could reflect a depletion of antioxidant capacity in prostate malignancy. Reduced ADH III activity may potentially impair the clearance of endogenous formaldehyde and other reactive aldehydes, leading to increased cellular damage and potentially contributing to mechanisms associated with tumor aggressiveness [39,40]. However, this observation is preliminary, as the difference was not statistically significant and aldehyde accumulation was not directly measured in the present study.

In our study, we observed significantly elevated ALDH class I and III activity in PCa tissue compared with BPH. ALDH is known for its role in metabolizing toxic aldehydes and maintaining cellular redox balance. Importantly, the fluorometric assay used in this study measures the combined activity of ALDH class I and III isoenzymes and does not allow differentiation between individual isoforms. Although both ALDH1A1 and ALDH3A1 have been reported to play important roles in prostate cancer, the present results cannot determine the contribution of either individual isoform to the observed increase in combined ALDH activity. ALDH activity has been proposed as a marker of cancer stem-like cells in multiple tumor types, including prostate cancer, and has been linked to treatment resistance and tumor aggressiveness [21,41]. The literature indicates that ALDH1A1 may also contribute to metastatic spread and resistance to radiotherapy, confirming that elevated ALDH activity reflects cellular properties associated with tumor survival and treatment resistance [41,42]. These metabolic changes can also affect the ability of cancer cells to survive in a microenvironment characterized by metabolic and oxidative stress [43]. Both ALDH1A1 and ALDH3A1 have been reported to play important roles in prostate cancer. ALDH1A1 expression has been associated with tumor-initiating cell properties and a poor prognosis, whereas ALDH3A1 has been linked to oxidative stress defence and drug resistance mechanisms [42,44]. The observed differences in ADH and ALDH activity may be more significant as indicators of metabolic adaptation and tumor biology than as standalone diagnostic biomarkers. In particular, elevated ALDH activity may reflect an enhanced capacity of cancer tissue to detoxify reactive aldehydes and cope with oxidative stress, thereby potentially promoting cancer cell survival. In prostate cancer, high ALDH1A1 expression was positively correlated with Gleason score and pathologic stage, and inversely associated with overall survival and cancer-specific survival of the patients [42]. Gorodetska et al. found that ALDH1A1 positively regulates tumor cell survival in circulation, extravasation, and metastatic dissemination through interactions with the androgen receptor [41]. Thus, the elevated ALDH activity observed in our study may be consistent with metabolic changes associated with tumor development and cellular stress adaptation, although the underlying mechanisms cannot be established from enzymatic activity measurements alone. However, given that this study assessed enzymatic activity in bulk tissue rather than analyzing protein expression of specific isoforms or treatment outcomes, the results should not be interpreted as evidence that ADH or ALDH activity can currently serve as a diagnostic or predictive biomarker. Further research could determine whether specific ADH/ALDH isoforms play a role in risk stratification, prognosis assessment, or the prediction of treatment response.

Our findings are broadly consistent with previous studies implicating ADH and ALDH enzymes in prostate cancer, although direct comparisons are limited by differences in the experimental material, analytical methods, and the specific isoenzymes investigated. The increased ALDH class I and III activity observed in both low- and intermediate-risk PCa groups’ tissues in our study may reflect early metabolic alterations associated with prostate cancer, although their biological significance cannot be determined from the present activity measurements alone. This difference should also be interpreted with caution because PCa and BPH tissues were obtained using different surgical procedures, and the potential effects of tissue handling, electrosurgical exposure, and other pre-analytical factors cannot be completely excluded. Therefore, the observed increase in ALDH activity cannot be attributed exclusively to malignant biology. Further studies using tissue samples obtained under comparable conditions will be needed to determine the biological significance of these findings. Our findings of elevated ALDH activity in prostate cancer tissue are consistent with previous reports showing that a subset of murine prostatic epithelial cells with high ALDH activity exhibits stem/progenitor-like characteristics. Moreover, consistent with our observations, clinical studies have identified ALDH-bright populations in freshly excised prostate cancer specimens, with ALDH1A1 expression significantly enriched in high Gleason tumors and advanced-stage disease. Importantly, ALDH1A1 positivity correlated with poor survival in hormone-naïve patients, highlighting the potential clinical relevance of ALDH1A1 reported in previous studies [45]. The correlation analysis revealed weak, statistically significant associations between several ADH isoenzymes, particularly between ADH I and ADH III and between ADH I and ADH IV. These correlations should be interpreted with caution because enzyme activities were normalized to protein concentration obtained from the same tissue supernatant, which may contribute to correlations between variables. Interestingly, no strong correlations were found between any ADH isoenzyme and ALDH, suggesting that these enzyme families may be regulated independently in prostate tissue or fulfill distinct biological roles.

Our results are consistent with previous studies showing altered ADH and ALDH activity in other malignancies. The analysis of enzymes that metabolize ethanol in various types of malignant tumors has been the subject of research for many years. This analysis has revealed significant changes in the activity of alcohol dehydrogenase and its isoenzymes, as well as aldehyde dehydrogenase, compared with healthy tissues. Jelski et al. reported a significant increase in total ADH activity in cancerous tissues in all gastrointestinal cancers studied, including esophageal, gastric, liver, pancreatic, and colorectal cancer, with class I or IV isoenzyme activity predominating in most cases [46,47,48,49,50]. Class IV ADH activity was particularly significant in the esophagus [46] and stomach [47], while class I dominated in the liver [48] and colon [49]. Meanwhile, ALDH activity in tumor tissues often remained unchanged or decreased, potentially resulting in the accumulation of toxic acetaldehyde. Similar changes were observed in gynecological cancers (endometrium, cervix, and ovary) [50,51,52,53], as well as in kidney [54] and brain cancer [55]. In the present study, however, increased ALDH class I and III activity was observed in PCa tissue, while the evidence for increased ADH activity was less consistent across isoenzymes. Therefore, the present findings do not support a simple model of increased acetaldehyde production without a corresponding increase in aldehyde detoxification. Although alterations in ADH and ALDH activities may indicate changes in aldehyde metabolism in prostate cancer, acetaldehyde accumulation or an imbalance between its production and degradation cannot be established from the present data, as acetaldehyde concentrations were not directly measured.

### Limitations and Future Directions

This study has some limitations. The relatively limited sample size, especially in subgroup comparisons (low vs. intermediate-risk PCa), due to the availability of eligible tissue specimens, may have reduced the statistical power to detect smaller differences between groups. Although enzymatic activity was measured directly, no parallel assessment of gene expression or protein levels was performed, which could further clarify regulatory mechanisms. Finally, the patients’ alcohol consumption history was not quantified in detail, although all participants reported occasional use, and the influence of ethanol exposure on ADH and ALDH activity cannot be excluded. Given the strong association between alcohol intake and prostate-related pathologies, as well as the potential for alcohol to directly or indirectly modulate ADH and ALDH activity, a more detailed stratification of patients according to alcohol consumption patterns would be valuable for improving the interpretation of enzymatic results in the case of a substantially larger study group. Another significant limitation is the lack of absolute substrate specificity in the enzymatic assays used, which could impact the interpretation of enzyme activity. The lack of mechanistic validation limits the biological interpretation of our findings. Changes in enzyme activity may be influenced by several factors, including differences in enzyme expression, post-translational regulation, or substrate availability. Therefore, our findings should primarily be interpreted as evidence of differences in enzymatic activity rather than as evidence of a specific molecular mechanism. Further studies assessing isoenzyme-specific gene and protein expression, together with functional validation, will be needed to better understand the mechanisms underlying the observed changes.

The use of BPH tissue as a control group is another limitation. Although obtaining completely healthy prostate tissue is difficult for both ethical and practical reasons, BPH itself may be accompanied by localized inflammation of varying severity, which could affect oxidative stress and enzyme activity. Therefore, the possible influence of inflammation should be considered when interpreting differences in enzymatic activity between BPH and PCa tissues.

An additional limitation is the difference in tissue sampling procedures between the PCa and BPH groups. PCa tissue was obtained during radical prostatectomy, whereas the BPH tissue was collected by transurethral electroresection. Whenever possible, tissue fragments from the central or deeper regions of the TUR specimens were selected to minimize the inclusion of visibly thermally affected tissue. Nevertheless, the potential influence of electrosurgical energy on enzyme activity cannot be completely excluded. Differences in tissue handling and exposure to irrigation fluid may also have contributed to pre-analytical variability. This is particularly relevant because enzyme activity was measured in crude tissue homogenates and expressed relative to total protein content. Consequently, differences in tissue integrity or protein recovery could potentially affect the measured activities. The observed differences between the PCa and BPH groups should therefore be interpreted with this potential source of pre-analytical variability in mind. Future studies should preferably use paired samples consisting of tumor tissue and adjacent histologically benign tissue obtained from the same radical prostatectomy specimen, which would substantially reduce the potential influence of differences in tissue sampling and handling.

A further limitation is that the biochemical analyses were performed on whole tissue fragments without quantitative histological assessment of tumor content. The analyzed samples may have contained different proportions of malignant epithelial cells, benign glands, and stromal tissue, which could have influenced the measured enzyme activity. Thus, the results reflect enzyme activity in the analyzed prostate tissue rather than activity specifically attributable to prostate cancer cells. Future studies combining biochemical analyses with histopathological assessment of tumor content and tissue composition, ideally using tumor-enriched material, would help to clarify the cellular and tissue sources of the observed changes in enzymatic activity.

Future studies should explore the molecular mechanisms underlying ADH, its isoenzymes, and ALDH regulation in prostate tissue, including isoform-specific expression, protein abundance, and associations with clinical outcomes. Combining these approaches with detailed histopathological characterization and molecular validation will be important for clarifying the mechanisms underlying the observed changes in enzymatic activity. Larger, multicenter studies involving independent patient populations will be needed to validate these findings, assess their reproducibility, and further evaluate their potential clinical relevance.

## 5. Conclusions

This study provides one of the first comprehensive functional analyses of both ADH and ALDH in prostate cancer tissue, addressing a notable gap in the literature. Our findings may reflect differences in the activity of ADH, its isoenzymes, and ALDH between prostate cancer and benign prostatic hyperplasia tissues. Increased activity of class I ADH and ALDH enzymes in prostate cancer tissue may reflect disease-associated metabolic changes and suggest a potential role of these enzymes in prostate cancer biology. These findings provide new insights into metabolic alterations in prostate cancer and support the need for further studies to clarify the mechanisms underlying the observed changes in enzymatic activity.

## Figures and Tables

**Table 1:** European Association of Urology risk groups for biochemical recurrence of prostate cancer [23].

Definition				
Low-Risk	Intermediate-Risk		High-Risk	
	Favourable	Unfavorable		
ISUP grade 1 and PSA < 10 ng/mL and cT1-2a *	ISUP grade 2 and PSA < 10 ng/mL and cT1-2b * Or ISUP grade 1 and PSA 10 –20 ng/mL and cT1-2b * Or ISUP grade 1 and PSA < 10 ng/mL and cT2b *	ISUP grade 2 and PSA 10–20 ng/mL and cT1-2b * Or ISUP grade 3 and cT1-2b *	ISUP grade 4/5 Or PSA > 20 ng/mL Or cT2c *	cT3-4 * and/or cN+ ** any ISUP grade * any PSA
Localized				Locally advanced

GS = Gleason score; ISUP = International Society for Urological Pathology; PSA = prostate-specific antigen. * Based on digital rectal examination. ** Based on CT/bone scan.

**Table 2:** ADH and ALDH activities in PCa and BPH tissues.

Enzyme	Group	Mean	Median	Min	Max	SD	Q1	Q3	95%CI (Median)	*p*-Value	r_rb
ADH I	PCa (*n* = 48)	0.072	0.060	0.009	0.229	0.045	0.048	0.082	0.044–0.076	0.049	0.227
	BPH (*n* = 42)	0.057	0.052	0.016	0.168	0.031	0.038	0.064	0.040–0.064		
ADH II	PCa (*n* = 48)	0.597	0.350	0.008	8.843	1.239	0.153	0.646	0.000–0.789	0.157	0.172
	BPH (*n* = 42)	0.359	0.246	0.013	1.780	0.418	0.109	0.405	0.088–0.404		
ADH III	PCa (*n* = 48)	7.659	6.174	0.107	28.457	6.307	3.083	11.782	3.938–8.410	0.186	0.160
	BPH (*n* = 42)	10.547	7.696	0.096	49.999	9.914	4.309	12.102	3.938–11.454		
ADH IV	PCa (*n* = 48)	5.472	3.609	0.054	35.369	7.177	1.041	6.458	1.064–6.154	0.151	0.175
	BPH (*n* = 42)	3.341	2.239	0.054	17.006	3.650	1.011	3.794	0.855–3.623		
Total ADH	PCa (*n* = 48)	0.642	0.558	0.001	1.800	0.435	0.362	0.918	0.404–0.712	0.449	0.092
	BPH (*n* = 42)	0.674	0.438	0.009	2.685	0.679	0.205	0.861	0.181–0.695		
ALDH I and III	PCa (*n* = 48)	0.378	0.242	0.001	2.150	0.461	0.089	0.327	0.079–0.405	0.0007	0.406
	BPH (*n* = 42)	0.259	0.103	0.007	4.668	0.719	0.053	0.199	0.000–0.376		

Units: nmol/min/mg of protein; SD—standard deviation; Q1—lower quartile, Q3—upper quartile; 95%CI—confidence intervals; r_rb-rank—biserial correlation; PCa—prostate cancer; BPH—benign prostatic hyperplasia; ADH—alcohol dehydrogenase; ALDH—aldehyde dehydrogenase.

**Table 3:** Spearman correlations between enzyme activities in PCa patients.

Total Study Group (*n* = 48)	ADH I	ADH II	ADH III	ADH IV	ADH Total	ALDH I and III
ADH I						
r		0.181	**0.317**	**0.305**	0.245	0.129
*p*		0.072	0.002 *	0.003 *	0.017 *	0.214
ADH II						
r	0.181		0.049	0.273	0.084	0.074
*p*	0.072		0.637	0.007 *	0.422	0.478
ADH III						
r	**0.317**	0.049		0.287	0.059	0.106
*p*	0.002 *	0.637		0.005 *	0.571	0.307
ADH IV						
r	**0.305**	0.273	0.287		0.084	0.137
*p*	0.003 *	0.007 *	0.005 *		0.421	0.190
ADH total						
r	0.245	0.084	0.059	0.084		0.073
*p*	0.017 *	0.422	0.571	0.421		0.481
ALDH I and III						
r	0.129	0.074	0.106	0.137	0.073	
*p*	0.214	0.478	0.307	0.190	0.481	

Correlation ratio (r): in bold, values in the range 0.301–0.700. *, significant correlation between tested variables. ADH-alcohol dehydrogenase; ALDH-aldehyde dehydrogenase; units for enzyme activity—nmol/min/mg of protein.

**Table 4:** Correlations between ADH activities and clinicopathological parameters in PCa patients.

Clinical Parameter	Enzyme Activity	*n*	Spearman’s r	*p*-Value
TNM stage	ADH I	48	0.040	0.787
TNM stage	ADH II	48	−0.203	0.167
TNM stage	ADH III	48	−0.195	0.185
TNM stage	ADH IV	48	0.153	0.299
TNM stage	Total ADH	48	0.092	0.534
TNM stage	ALDH I + III	48	−0.219	0.135
Gleason score	ADH I	48	−0.116	0.428
Gleason score	ADH II	48	−0.076	0.604
Gleason score	ADH III	48	−0.374	0.008
Gleason score	ADH IV	48	−0.076	0.606
Gleason score	Total ADH	48	0.023	0.876
Gleason score	ALDH	48	0.037	0.803
PSA	ADH I	48	0.056	0.701
PSA	ADH II	48	0.107	0.464
PSA	ADH III	48	−0.040	0.782
PSA	ADH IV	48	−0.020	0.891
PSA	Total ADH	48	0.122	0.404
PSA	ALDH I + III	48	0.188	0.195

## Data Availability

The raw data supporting the conclusions of this article will be made available by the authors on request.
